# *Decaisninatomentosa* (Loranthaceae), a new species of mistletoe from Samar Island, Philippines

**DOI:** 10.3897/BDJ.10.e78457

**Published:** 2022-03-01

**Authors:** Danilo N. Tandang, Marjorie delos Angeles, Inocencio, Jr. E. Buot, Mohan Prasad Devkota, Marcos A. Caraballo-Ortiz

**Affiliations:** 1 Philippine National Herbarium, Botany and National Herbarium Division, National Museum of Natural History, National Museum of the Philippines, T.M. Kalaw St., Ermita Manila 1000, Metro Manila, Philippines Philippine National Herbarium, Botany and National Herbarium Division, National Museum of Natural History, National Museum of the Philippines, T.M. Kalaw St., Ermita Manila 1000 Metro Manila Philippines; 2 Biodiversity Program, Taiwan International Graduate Program, Academia Sinica and National Taiwan Normal University, Taipei, Taiwan Biodiversity Program, Taiwan International Graduate Program, Academia Sinica and National Taiwan Normal University Taipei Taiwan; 3 Department of Life Science, National Taiwan Normal University, Taipei 11677, Taipei, Taiwan Department of Life Science, National Taiwan Normal University, Taipei 11677 Taipei Taiwan; 4 Biodiversity Research Center, Academia Sinica, Taipei 11529, Taipei, Taiwan Biodiversity Research Center, Academia Sinica, Taipei 11529 Taipei Taiwan; 5 Center for Integrative Conservation, Xishuangbanna Tropical Botanical Garden, Chinese Academy of Sciences, CN-666303 Menglun, Yunnan, China Center for Integrative Conservation, Xishuangbanna Tropical Botanical Garden, Chinese Academy of Sciences, CN-666303 Menglun Yunnan China; 6 University of the Philippines Los Baños, Laguna, Philippines University of the Philippines Los Baños Laguna Philippines; 7 Plant Biology Division, Institute of Biological Sciences, College of Arts and Sciences, University of the Philippines Los Baños, Laguna, Philippines Plant Biology Division, Institute of Biological Sciences, College of Arts and Sciences, University of the Philippines Los Baños Laguna Philippines; 8 Tribhuvan University, Kathmandu, Nepal Tribhuvan University Kathmandu Nepal; 9 Smithsonian Institution, Washington, DC, United States of America Smithsonian Institution Washington, DC United States of America

**Keywords:** forest over limestone, island endemics, parasitic plants, Samar Island Natural Park, Santalales, taxonomy

## Abstract

**Background:**

The extensive forests over limestone of the Samar Island Natural Park (SINP) on Samar Island, Philippines harbour a rich variety of unique species. In this contribution, we describe and illustrate a new Loranthaceous mistletoe, endemic to Samar Island named *Decaisninatomentosa*, representing the 12th member of the genus reported to the Philippines.

**New information:**

This new species is similar to *D.confertiflora* (Merr.) Barlow with respect to the presence of shortly and densely off-white tomentose inflorescence and 6-merous flowers. However, it is unique amongst congeners in having tomentose and partially fused bracteoles which cover almost or entirely the ovary of individual flowers in the triads. To date, *D.tomentosa* seems to be restricted to the SINP and is only known from a handful of individuals. More studies are needed to properly assess the demography, host range, conservation status and phylogenetic position of this distinctive species of mistletoe.

## Introduction

The Samar Island Natural Park (SINP) in Samar Island, Philippines is one of the largest contiguous old-growth forests in the country, covering approximately 333,300 hectares of mostly lowland dipterocarp rainforest and forest over limestone (Mallari et al. 2001, UNDP [United Nations Development Programme] 2007, Taylor et al. 2015). Due to its unique geomorphological karstic features, forests over limestones are areas of great biological importance worldwide with high endemicity and unique assemblages of flora and fauna (Fernando et al. 2008, Struebig et al. 2009).

Recent botanical explorations of forest over limestone in the SINP revealed a mistletoe that did not fit with any of the currently-described species reported for the Philippines. A detailed examination of its morphological features placed it within *Decaisnina* Tiegh (1895: 435) by Tieghem (1895), a genus with over 25 species distributed from northern Australia to the Eastern Malay Archipelago (including Java, Celebes and the Philippines) and reaching the Marquesas (Barlow 1997, POWO 2021). *Decaisnina* are aerial hemi-parasitic shrubs within the Showy Mistletoe family (Santalales, Loranthaceae) (Barlow 1993, Barlow 1997, Tandang et al. 2021). Inflorescence of this family can be axillary or terminal, racemes, spikes or umbels (Huaxing and Gilbert 2003).

In the Philippines, *Decaisnina* follows *Amyema* Tiegh (1894: 506) as the second most numerous genus of Loranthaceae with 11 species, eight of them endemic (Pelser et al. 2011). In the Visayas Region, where the SINP is located, the following species of *Decaisnina* have been reported (Pelser et al. 2011): *D.aherniana* (Merr.) Barlow (1993: 74), *D.confertiflora* (Merr.) Barlow (1993: 80), *D.crassilimba* (Merr.) Barlow (1993: 82), *D.cumingii* (Tiegh.) Barlow (1983: 82), *D.ovatifolia* (Merr.) Barlow (1993: 90) and *D.sumbawensis* (Tiegh.) Barlow (1993: 95). In June 2021, M.D. delos Angeles collected a *Decaisnina* with a combination of morphological characters that differs from all the previously-documented species for the country. After a thorough review of published literature and comparisons with online photographs of other mistletoes available at Phytoimages (Nickrent et al. 2006) and Co’s digital flora of the Philippines (Pelser et al. 2011), we consider that this specimen represents an undescribed taxon. Hence, here we describe it as a new species, representing the 12th species of *Decaisnina* reported to the Philippines.

## Materials and methods

We conducted a field survey at the SINP in June 2021 aiming to document the regional flora along the eco-trail. We observed five individuals of an undescribed *Decaisnina* species at the site and took photographs of their habit and other important features from fresh material. The holotype and isotype specimens were prepared from a single individual. The species description was constructed by combining observations of living structures studied at the type locality along with measurements of material preserved in alcohol made at the Institute of Biological Sciences, College of Art and Sciences, University of the Philippines Los Baños. Measurements of morphological traits and anatomical features from the alcohol-preserved tissue were done under a stereomicroscope using a ruler and a digital caliper (150 mm; #245111, Tactix, Shanghai, China). After studying the material, specimens were processed into vouchers following standard protocols. The holotype was deposited at the Philippine National Herbarium (PNH), while the isotype was deposited at the College of Agriculture Herbarium UP (CAHUP) (acronyms follow Thiers 2021). Morphological data from closely related species used for comparisons were obtained from published literature.

In spite of conducting three separate expeditions during the months of June, September and October (~ 20 days) to the SINP from Paranas to Taft,covering approximately 30 km^2^ of terrain searching for more populations of the undescribed *Decaisnina*, we could not locate more individuals. We acknowledge that the lack of additional individuals limits our ability to assess the range of morphological variation within this species. However, the key characters separating *D.tomentosa* from related taxa were present in all of the individuals observed, suggesting that these features are consistent and, thus, of taxonomic value. The recognition of this new species follows the morphological-phenetic species concept, which clusters entities sharing a series of morphological characters and separate them from others by consistent morphological gaps (Judd 2007). Given the current lack of genomic data for the new species, we are unable to test concepts on phylogenetic classifications, but future molecular studies should infer its evolutionary history to assess relationships and confirm its uniqueness from a phylogenetic perspective.

## Taxon treatments

### Decaisnina tomentosa

M.D.Angeles, Tandang, Carab.-Ort. & Buot sp. nov.

CB6BBFC6-6BB8-5959-A21C-4B211F449018

#### Materials

- **Type status:** Holotype. **Occurrence:** catalogNumber: PNH 258558; recordedBy: Marjorie delos Angeles; individualCount: 1; lifeStage: mature; occurrenceID: urn:catalog:PNH:Plants:258558; **Taxon:** scientificName: *Decaisninatomentosa*; family: Loranthaceae; genus: Decaisnina; specificEpithet: *tomentosa*; scientificNameAuthorship: M.D.Angeles, Tandang, Carab.-Ort. & Buot; **Location:** country: Philippines; stateProvince: Samar; locality: Paranas, Samar Island Natural Park; verbatimElevation: 100 m; locationRemarks: label transliteration: "Samar Island Natural Park, 2021.06.03, Marjorie delos Angeles"; 100 m, 12°3’59”N 125°27’11”E, 2021.06.03, purposive sampling; verbatimCoordinates: 12°3’59”N 125°27’11”E; decimalLatitude: 12.359; decimalLongitude: 125.2711; georeferenceProtocol: label; **Identification:** identifiedBy: Marjorie D. delos Angeles and Danilo N. Tandang; dateIdentified: 2021; **Event:** samplingProtocol: purposive sampling; eventDate: 03/06/2021; **Record Level:** language: en; collectionCode: Plants; basisOfRecord: PreservedSpecimen
- **Type status:** Isotype. **Occurrence:** catalogNumber: CAHUP 074155; recordedBy: Marjorie delos Angeles; individualCount: 1; lifeStage: mature; occurrenceID: urn:catalog:CAHUP:Plants:074155; **Taxon:** scientificName: *Decaisninatomentosa*; family: Loranthaceae; genus: Decaisnina; specificEpithet: *tomentosa*; scientificNameAuthorship: M.D.Angeles, Tandang, Carab.-Ort. & Buot; **Location:** country: Philippines; stateProvince: Samar; locality: Paranas, Samar Island Natural Park; verbatimElevation: 100 m; locationRemarks: label transliteration: "Samar Island Natural Park, 2021.06.03, Marjorie delos Angeles"; 100 m, 12°3’59”N 125°27’11”E, 2021.06.03, purposive sampling; verbatimCoordinates: 12°3’59”N 125°27’11”E; decimalLatitude: 12.359; decimalLongitude: 125.2711; georeferenceProtocol: label; **Identification:** identifiedBy: Marjorie D. delos Angeles and Danilo N. Tandang; dateIdentified: 2021; **Event:** samplingProtocol: purposive sampling; eventDate: 03/06/2021; **Record Level:** language: en; collectionCode: Plants; basisOfRecord: PreservedSpecimen

#### Description

Climbing aerial stem-parasitic shrubs ca. 30 cm in height; epicortical roots present, terete, glabrous, 10 cm long, stout at base, 0.8 cm becoming slender, 0.5 towards the apex, haustoria at irregular intervals, appressed against the stem of the host tree. **Stems** with internodes somewhat flat on younger twigs, becoming terete, 3.8–5.9 × 0.2–0.7 cm, smooth when bearing young leaves, coarsely and irregularly ridged when older, light brown to greyish-white; nodes dilated on matured stems. **Leaves** opposite; adaxial surface dark green and glossy, abaxial surface pale and dull, glabrous, concave, lanceolate to broadly ovate, 6.3–9.6 × 3.1–5.7 cm; margin entire; apex acute to attenuate; base cuneate, decurrent towards a channelled petiole, especially in young leaves; petioles terete, 3.4–7.8 × 2.3–2.6 mm, greyish-white on older branches, light green and almost sessile on young twigs, 2.2–3.6 × 1.2–1.8 mm; veins with mid-rib rarely pale green at base on adaxial surface, raised only on the abaxial surface, secondary venation pinnate and obscure on both sides. **Inflorescences** absence of involucre of decussate scales, axillary on leafless nodes; short, unbranched raceme with 5–7 opposite pairs of triads with flowers that are sessile 0.4–0.9 × 0.9–1.2 mm, bract at the base of the inflorescence absent, tomentose all throughout, except for the inner lobes of bracteoles and limb, stamen and pistil; thyrse 16.2–35 mm long, 2.1–2.6 mm wide at base, 1.3–1.8 mm wide towards the apex, terete; inflorescence internodes 1.9–4.9 × 0.5–1.3 mm; peduncle of triads terete 5.2–8.3 ×1.0–1.3 mm, secund; floral bracteoles partially fused forming a cup, 4.0–4.1 × 3.6–3.7 mm, united below the middle, cucullate, lobes of bracteoles partly overlapping at base, margin tomentose, apex obtuse, tomentose outside, glabrous inside, enclosing the ovary; ovary white, widely obovate, 2.8–2.9 × 1.4–1.5 mm; calyculus narrowly cylindrical, 0.8–1.1 × 1.3–1.2 mm, limb erect, cupular, irregularly lobed, apex acute to aristate, glabrous inside. **Flowers** 6-merous with corolla bud of variable colours, red or red orange at base, green at apex; mature buds usually basally red, green at tip, 26.1–29.5 × 2.3–2.5 mm long, clavate, length of corolla tube at anthesis 2.1–3.4 × 1.7–2.1 mm, becoming constricted, 2.3–3.8 × 1–1.2 mm and inflated, 3.7–3.9 × 1.7–2.4 mm, tubular and obtuse or rounded at tip, tomentose; petal lobes 11.3–15.8 mm long, broad at base 1.2–1.4 mm, middle part 0.6–0.8 mm, near apex 0.5–1 mm, coherent in the second inflated part 9–11 mm in height from base, recurved petal lobe 6.4–6.9 mm, acute to rounded at apex; stamens 25.3–27.8 × 0.2–0.3 mm; anthers 2.9–3.6 mm long, basifixed, more or less arranged at the same level, acuminate at apex, longer than the free part of the filament; disc 0.6 × 0.5 mm wide; style 28.6–29.1 × 0.3–0.4 mm long; stigma 0.27–0.29 × 0.2–0.3 mm, circular to elliptic. **Fruit** ovoid, 5.7–6.7 × 4.2–4.4 mm, ripening yellow-green, calyculus persistent; viscin disc 1.6–2.1 mm thick. **Seeds** 4.2–4.4 × 1.7–1.8 mm, white.

#### Diagnosis

**Figure 1.**
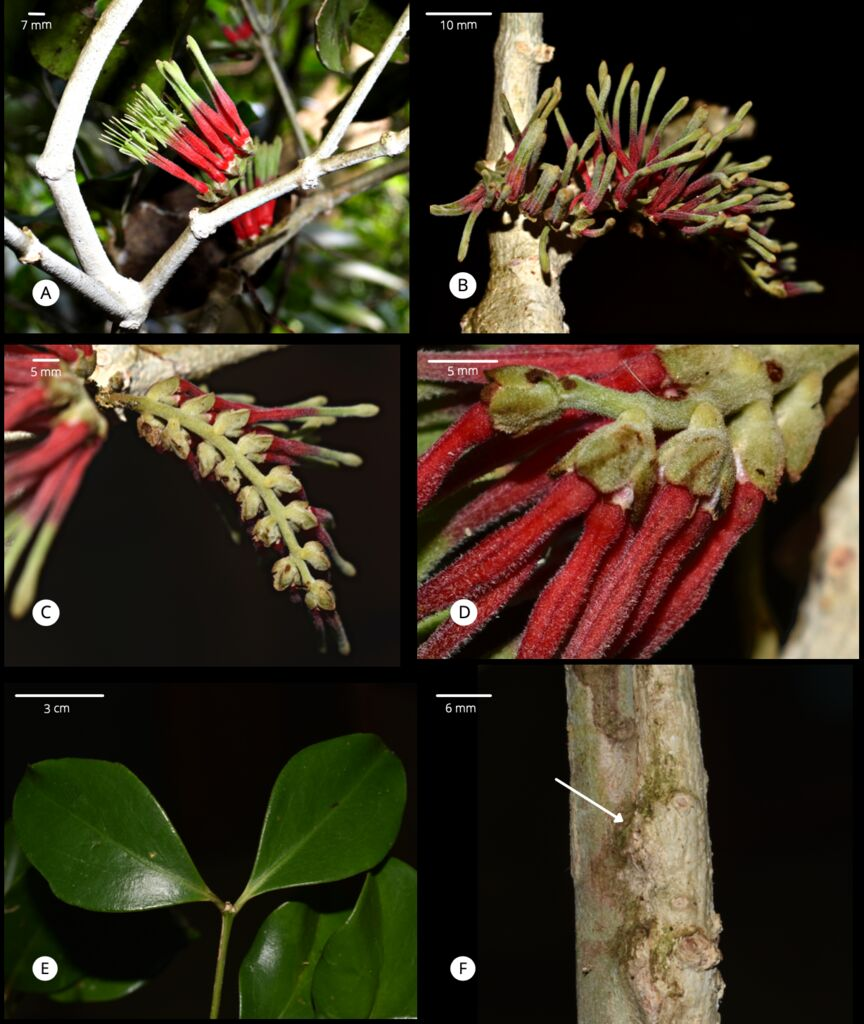
*Decaisninatomentosa* M.D.Angeles, Tandang, Carab.-Ort., & Buot. **A** Inflorescence with distal flowers in buds and proximal flowers at anthesis. **B** Inflorescence with flower buds. **C** Inflorescence in a secund raceme showing the tomentose rachis and bracts. **D** Close-up of bracts and bracteoles showing its tomentose nature. Note the tomentose indument covering all structures. **E** Twig with opposite leaves. Note the shiny surface of leaves with obscure venation. **F** Epicortical runner (right side) appressed against the stem of its host. Arrow points to a secondary haustorial connection. Photo credits: Marjorie delos Angeles.

*Decaisninatomentosa* (Fig. 1) is similar to *D.confertiflora* (Merr.) Barlow with respect to the presence of shortly and densely off-white tomentose inflorescence and 6-merous flowers. However, the new species differs by its rounded ste, (*vs.* stem internode distinctly angular), cuneat leaf base (*vs.* truncate or slightly cordate at the leaf base) and corolla that is distinctly inflated teice (*vs.* slightly inflated at the base). Also, the new species has a longer corolla measuring 27.9–*29.2 mm (vs. 16–20 mm). Highly distinct flowers that are subtended with a tomentose cup formed by the fusion of the bracteoles with slightly cucullate lobes and glabrous inside which almost or entirely cover the ovary of individual flowers, and corolla tomentose, double inflated at base and middle.* We consider that the combination of these and other characters presented in Table 1 are significant for the recognition of this new species.

#### Etymology

The specific epithet “tomentosa” refers to the short soft indument densely covering the inflorescence of the new species.

#### Distribution

This new species is known only from the type locality at the SINP in Paranas, Samar Island. The SINP is a protected area under Proclamation No. 442. 2003, pursuant the National Integrated Protected Areas System Act of 1992 (Republic Act No. 7586).

#### Ecology

Regarding interactions with potential pollinators and seed dispersers, no floral visitors or fruit consumers were observed during our visit to the site. Given the morphological features of flowers and fruits, it is probable that they are pollinated and dispersed by birds. Systematic field observations and experimental studies are needed to assess pollinators and effective seed dispersers and to document interactions with other organisms.

#### Conservation

During the first field survey in June 2021, we observed a tree of *Baccaureaphilippinensis* Merr. (1915: 275) (Phyllanthaceae) parasitised by five individuals of *D.tomentosa*. Searches for additional individuals in the area were unfruitful. Two additional explorations to the SINP in September and October 2021 failed to locate more individuals. Since *D.tomentosa* is only known from five individuals on a single tree, we believe that the species is vulnerable to extinction. However, we need information from additional surveys to identify more individuals and perform quantitative assessments on its demographic profile, current distribution, host range and potential threats. Therefore, we consider that the species should be listed as Data Deficient (DD) (IUCN Standards and Petitions Subcommittee 2019) until more field assessments are conducted to ascertain its actual conservation status.

#### Biology

*Decaisninatomentosa* has been observed bearing flowers and fruits in the month of June.

#### Notes

*Decaisninatomentosa* resembles the widespread species complex *D.sumbawensis* (Tiegh.) Barlow (1997: 304) due to morphological resemblances in the colour of the mature corolla (basally red and apically green), but the former can be separated from the latter by the presence of densely covered short soft hairs on the inflorescence (tomentose vs. puberulent).

## Supplementary Material

XML Treatment for Decaisnina tomentosa

## Figures and Tables

**Table 1.** Table comparing morphological characters between *Decaisninatomentosa* M.D.Angeles, Tandang, Carab.-Ort., & Buot and *D.confertiflora* (Merr.) Barlow were obtained from Barlow (1997).

**Character**	***Decaisninatomentosa***	***Decaisninaconfertiflora***
Stem		
Shape	Terete	Distinctly 4-angular
Leaves		
Dimension (cm)	6–10 × 3–6	12–20 × 5–8
Base	Cuneate	Truncate or slightly cordate
Inflorescence		
Triad	5–7 pairs	10–15 pairs
Peduncle of triads (mm)	5.2–8.3	1–2
Flowers		
Corolla	Distinctly inflated twice	Slightly inflated at the base
Corolla length (mm)	27.9–29.2	16–20
Petals in open flower	Coherent in the lower 9–11 mm	
Anther (mm)	2.9–3.6 mm long, acute, longer than the free part of the filament	ca. 2
